# Autophagy in inflammation: the p38α MAPK-ULK1 axis

**Published:** 2018-03-09

**Authors:** Hua She, Yingli He, Yingren Zhao, Zixu Mao

**Affiliations:** 1 Department of Pharmacology, Emory University School of Medicine, 615 Michael Street, Atlanta, GA 30322 USA; 2 Department of Neurology, Emory University School of Medicine, 615 Michael Street, Atlanta, GA 30322 USA; 3 Department of Infectious Diseases, The First Affiliated Hospital, Xi’an Jiaotong University School of Medicine, Xi’an, Shaanxi, 710000 China

**Keywords:** autophagy, p38α MAPK, ULK1, inflammation

## Abstract

Autophagy and inflammation are two processes vital for immune cells to perform their functions. Their proper interplay upon signal is pivotal for proper response to stress. The stress kinase p38α MAPK in microglia senses inflammatory cue LPS, directly phosphorylates ULK1, relieves the autophagic inhibition on the inflammatory machinery, and thus allows for a full immune response.

Autophagy and inflammation are two fundamental biological processes that are involved in both physiological and pathophysiological conditions ^[1, 2]^. Through its role in maintaining cellular homeostasis by disposal of damaged organelles, aggregated proteins, as well as invaded pathogens via a lysosomal degradation pathway, autophagy is involved in the modulation of cell metabolism, host defense, and cell survival. Defective autophagy is commonly associated with pathological conditions such as inflammation and autoimmune diseases, neurodegenerative diseases as well as aging ^[3, 4]^. Besides autophagy, the cellular response to stress involves numerous other pathways, of which, the most common and important is inflammation. Innate immune cells respond to endogenous or exogenous irritations and injuries. The inflammatory response can be either protective or destructive depending on the context of the insults and the stage of the response ^[5]^.

Recent studies have highlighted the cross-talk between autophagy and inflammation. Increasing evidences show that autophagy plays important roles in both innate and adaptive immunity through eliminating invading pathogens, regulating innate pathogen recognition, contributing to antigen presentation via major histocompatibility complex class I/II molecules, and controlling B cell and T cell development and survival ^[6, 7]^. It is also becoming increasingly clear that immune signaling cascades are subject to regulation by autophagy, and a return to homeostasis following a robust immune response is critically dependent on autophagy. Autophagy dysfunction contributes to the pathogenesis of various inflammation-related disorders ^[8]^. On the other hand, more and more studies indicate that a variety of immune mediators either induce or repress autophagy. For example, it is well established that in general Th1 cytokines, including IFN-γ, TNF-α, IL-1, IL-2, IL-6 and TGF-β, induce autophagy while the classical Th2 cytokines, including IL-4, IL-10 and IL-13, have the effects of autophagy inhibition ^[9]^.

Lipopolysaccharide (LPS), a gram-negative bacteria outer-wall component, has been shown to inhibit autophagy and induce microglia activation through binding to its cognate receptor complex-Toll like receptor 4 (TLR4) on microglia surface ^[6]^. However, the signaling mechanisms that lead to LPS-induced autophagy reduction and whether such a reduction is required for activating inflammation in microglia remain unknown. The stress kinase *p38α* mitogen-activated protein kinase (p38α MAPK) plays a central role in inflammation and is the master kinase for activation of *NOD-like receptor protein 3* (NLRP3) inflammasome in microglia ^[10]^. The p38α MAPK has been the subject of extensive efforts in both basic research and drug discovery for the treatment of a wide range of diseases. Inhibitors of p38 MAPK are currently in development for the clinical trial for several inflammatory diseases such as *Crohn’s* disease and rheumatoid arthritis ^[11, 12]^. Also of note, a key initial event in autophagy is the formation of the autophagosome, a unique double-membrane organelle that engulfs the cytosolic cargo destined for degradation. This step is mediated by the serine/threonine protein kinase unc-51-like kinase 1 (ULK1), which functions in a complex with at least three other protein partners, focal adhesion kinase family interacting protein of 200 kDa (FIP200), autophagy-related protein 13 (ATG13), and ATG101. A plethora of different upstream pathways, such as nutrients sensing by AMPK and mTOR, converge on ULK1, suggesting that this complex acts as a signaling node and convert multiple cellular inputs into tight regulation of autophagosome formation^[13, 14]^.

Our recent work showed that p38α MAPK plays a direct and essential role in relieving the inhibitory autophagic controlling of inflammation in response to inflammatory signals^[15]^. We found that p38α MAPK interacts with ULK1 in microglia. Upon LPS stimulation of TLR4 on microglia surface, activated p38α MAPK directly phosphorylates ULK1, the serine/threonine kinase in the initiation complex of the autophagic cascade, in primary microglia and in animal brain. Phosphorylation by p38α MAPK inhibits ULK1 kinase activity and disrupts its interaction with a key partner ATG13 in the autophagy initiation complex, and reduces the level and flux of autophagy. This p38α MAPK/ULK1-induced autophagy inhibition is necessary for LPS-induced NLRP3 inflammasome activity, subsequent processing of pro-*interleukin-1β (pro*-IL-1β) into IL-1β by caspase-1, and microglia full activation in culture and in mouse brain (Figure 1). Thus, our findings establish a mechanism that functions to relieve the immune suppressive activity of autophagy upon stimulation and allows the full induction of inflammatory process during microglial activation. This mechanism may play an important role in regulating innate immune response in the central nervous system.

It should be noted that many previous studies have reported that LPS-induced macrophage activation and secretion of inflammatory cytokines/chemokines is accompanied by enhanced autophagy activity ^[16]^. In addition, this is also mediated through TLR4 and p38 MAPK as inhibition of either TLR4 or p38 MAPK blocks LPS-induced autophagy increase and macrophage activation ^[17, 18]^. This is in clear contrast to the finding that autophagy is significantly reduced under both acute and chronic inflammatory conditions in microglia. For example, induction of autophagy activity by rapamycin has been shown to inhibit microglia over-activation, reduce the secretion of pro-inflammatory mediators, and provide protection against various insults in several animal models of neurodegenerative diseases including *Alzheimer’s disease* and *Parkinson’s disease* ^[19]^. It seems that autophagy plays different roles in macrophage and microglia in their inflammatory response. The molecular mechanisms underlie this sharp difference warrants further investigation. In our work, we notice that in microglia cell line BV2 cells, LPS-induced p38α MAPK activation leads to a strong ULK1 phosphorylation. But in macrophage derived RAW264.7 cells, p38α MAPK activity and ULK1 phosphorylation appear to be uncoupled. In addition, previous report showed that LPS induces a robust Nrf2-dependent transcription of p62, a key autophagy adaptor protein, in macrophage ^[20]^. While under our experimental condition, LPS has no effect on the transcription of p62 in microglia. These results further highlight the inherent differences among immune cells derived from different origins.

Accumulating evidence suggests that microphage and microglia acquire different activation states to modulate their cellular functions under different contexts ^[21]^. Upon activation to the M1 phenotype, macrophage and microglia release pro-inflammatory cytokines and neurotoxic molecules promoting inflammation and cytotoxic responses. In contrast, when adopting the M2 phenotype, they secrete anti-inflammatory gene products and trophic factors that promote repair and regeneration to restore homeostasis ^[22]^. The M1 and M2 states can coexist at the same time in different population of cells around the same lesion site. In addition, under certain situations, a single cell can display both M1 and M2 phenotypes simultaneously. Intriguing and challenging questions are how these apparent opposite activation states are regulated and whether p38α MAPK-ULK1 axis plays a role during the switch of the two states in microglia. Furthermore, very little is known about the switch of microglia activation state during disease progress. A better understanding is essential for developing more efficient protective agents. p38α MAPK-ULK1 axis may offer a new target to modulate microglia activation state and suppress their deleterious pro-inflammatory neurotoxicity as a therapeutic approach for the treatment of inflammatory and neurodegenerative diseases.

In conclusion, autophagy and inflammation are two key intertwined cellular processes that act together to modulate functions of innate immune cells. Their interplay may be distinctly regulated in microglia and macrophage. A better understanding of the regulatory mechanism of immune cell activation should provide insight for designing more sensible therapeutic strategies for the many immune-related diseases.

## Figures and Tables

**Figure 1:**
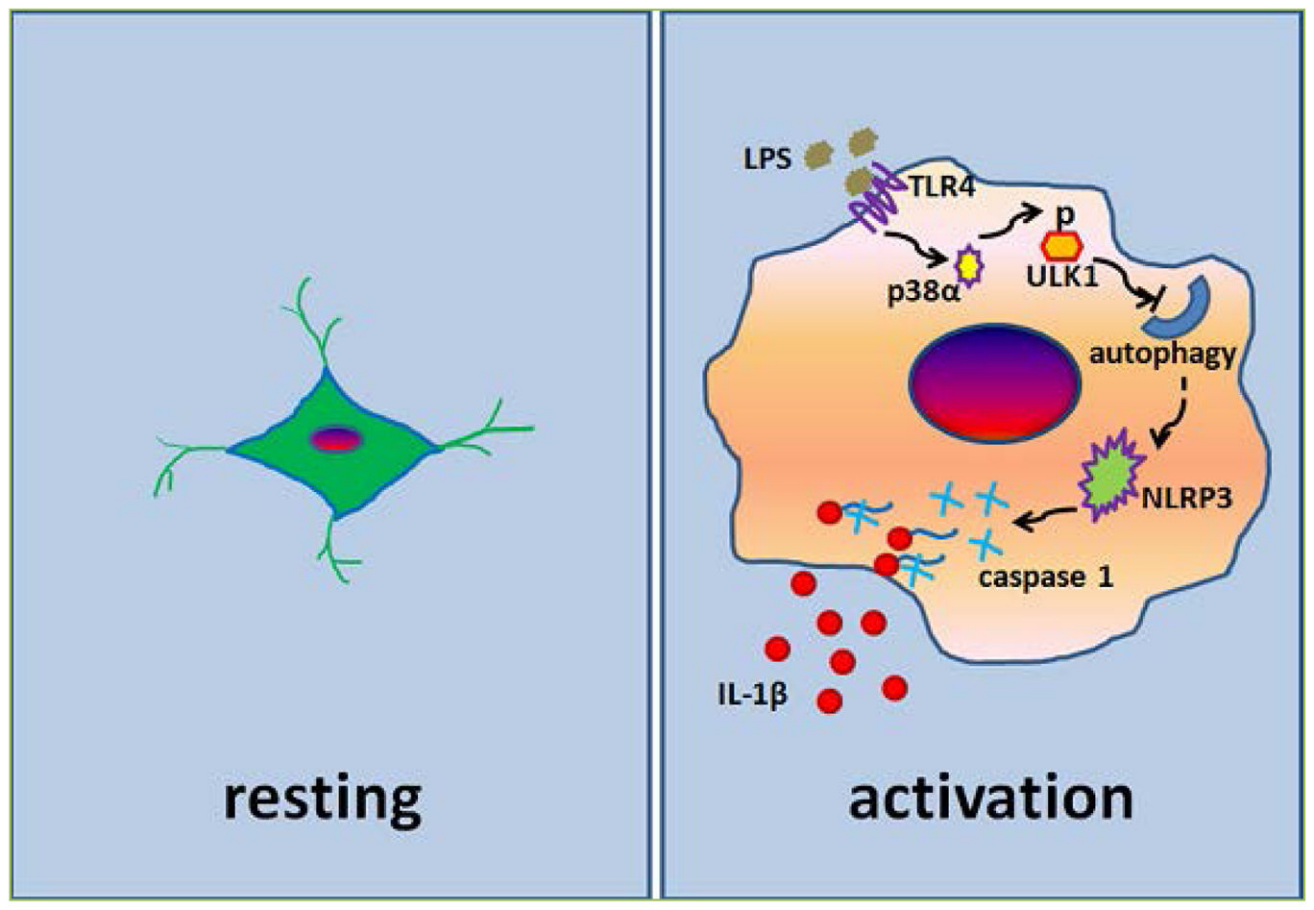
Regulation of inflammation through the p38α MAPK-ULK1 axis in microglia The resting microglial cell is characterized by a small cell body and much ramified thin processes, which extend in multiple directions (left). LPS binds to TLR4 and triggers p38α MAPK-dependent phosphorylation of ULK1 in microglial cells. This phosphorylation inhibits ULK1 kinase activity and reduces autophagy in microglia. Reduced autophagy activity activates NLRP3 inflammasome and leads to caspase 1 dependent production of IL-1β and microglia morphologic changes (right).

## Abbreviations

p38α MAPK: *p38α* mitogen-activated protein kinase
ULK1: unc-51-like kinase 1
LPS: lipopolysaccharide
TLR4: Toll like receptor 4
NLRP3: *NOD-like receptor protein 3*
ATG13: autophagy-related protein 13
IL-1β: *interleukin-1β.*

## References

[1] Netea-Maier RT, Plantinga TS, van de Veerdonk FL, Smit JW, Netea MG (2016). Modulation of inflammation by autophagy: Consequences for human disease. Autophagy.

[2] Shibutani ST, Saitoh T, Nowag H, Munz C, Yoshimori T (2015). Autophagy and autophagy-related proteins in the immune system. Nat Immunol.

[3] Zhong Z, Sanchez-Lopez E, Karin M (2016). Autophagy, Inflammation, and Immunity: A Troika Governing Cancer and Its Treatment. Cell.

[4] Cuervo AM, Macian F (2014). Autophagy and the immune function in aging. Curr Opin Immunol.

[5] Takahama M, Akira S, Saitoh T (2018). Autophagy limits activation of the inflammasomes. Immunol Rev.

[6] Puleston DJ, Simon AK (2014). Autophagy in the immune system. Immunology.

[7] Zhang H, Puleston DJ, Simon AK (2016). Autophagy and Immune Senescence. Trends Mol Med.

[8] Cadwell K (2016). Crosstalk between autophagy and inflammatory signalling pathways: balancing defence and homeostasis. Nat Rev Immunol.

[9] Qian M, Fang X, Wang X (2017). Autophagy and inflammation. Clin Transl Med.

[10] Hoseini Z, Sepahvand F, Rashidi B, Masoudifar A, Mirzaei H (2018). NLRP3 inflammasome: Its regulation and involvement in atherosclerosis. J Cell Physiol.

[11] Schieven GL (2005). The biology of p38 kinase: a central role in inflammation. Curr Top Med Chem.

[12] Schieven GL (2009). The p38alpha kinase plays a central role in inflammation. Curr Top Med Chem.

[13] Lin MG, Hurley JH (2016). Structure and function of the ULK1 complex in autophagy. Curr Opin Cell Biol.

[14] Zachari M, Ganley IG (2017). The mammalian ULK1 complex and autophagy initiation. Essays Biochem.

[15] He Y, She H, Zhang T, Xu H, Cheng L, Yepes M (2018). p38 MAPK inhibits autophagy and promotes microglial inflammatory responses by phosphorylating ULK1. J Cell Biol.

[16] Levine B, Mizushima N, Virgin HW (2011). Autophagy in immunity and inflammation. Nature.

[17] Xu Y, Jagannath C, Liu XD, Sharafkhaneh A, Kolodziejska KE, Eissa NT (2007). Toll-like receptor 4 is a sensor for autophagy associated with innate immunity. Immunity.

[18] Bode JG, Ehlting C, Haussinger D (2012). The macrophage response towards LPS and its control through the p38(MAPK)-STAT3 axis. Cell Signal.

[19] Su P, Zhang J, Wang D, Zhao F, Cao Z, Aschner M (2016). The role of autophagy in modulation of neuroinflammation in microglia. Neuroscience.

[20] Fujita K, Maeda D, Xiao Q, Srinivasula SM (2011). Nrf2-mediated induction of p62 controls Toll-like receptor-4-driven aggresome-like induced structure formation and autophagic degradation. Proc Natl Acad Sci U S A.

[21] Amici SA, Dong J, Guerau-de-Arellano M (2017). Molecular Mechanisms Modulating the Phenotype of Macrophages and Microglia. Front Immunol.

[22] Subramaniam SR, Federoff HJ (2017). Targeting Microglial Activation States as a Therapeutic Avenue in Parkinson’s Disease. Front Aging Neurosci.

